# Ablation of lysozyme M-positive cells prevents aircraft noise-induced vascular damage without improving cerebral side effects

**DOI:** 10.1007/s00395-021-00869-5

**Published:** 2021-04-30

**Authors:** Katie Frenis, Johanna Helmstädter, Yue Ruan, Eva Schramm, Sanela Kalinovic, Swenja Kröller-Schön, Maria Teresa Bayo Jimenez, Omar Hahad, Matthias Oelze, Subao Jiang, Philip Wenzel, Clemens J. Sommer, Katrin B. M. Frauenknecht, Ari Waisman, Adrian Gericke, Andreas Daiber, Thomas Münzel, Sebastian Steven

**Affiliations:** 1 Department of Cardiology, Cardiology I—Laboratory of Molecular Cardiology, University Medical Center of the Johannes Gutenberg-University, Building 605, Langenbeckstr. 1, 55131 Mainz, Germany; 2 Department of Ophthalmology, University Medical Center of the Johannes Gutenberg University, Mainz, Germany; 3 Institute for Molecular Medicine, University Medical Center of the Johannes Gutenberg-University Mainz, Mainz, Germany; 4 German Center for Cardiovascular Research (DZHK), Partner Site Rhine-Main, Mainz, Germany; 5 Center for Thrombosis and Hemostasis, University Medical Center of the Johannes Gutenberg-University, Mainz, Germany; 6 Institute of Neuropathology, University Medical Center of the Johannes Gutenberg-University, Mainz, Germany

**Keywords:** Environmental risk factor, Aircraft noise exposure, Oxidative stress, Endothelial dysfunction, Myeloid cell ablation, Cerebral inflammation, Diphtheria toxin

## Abstract

Aircraft noise induces vascular and cerebral inflammation and oxidative stress causing hypertension and cardiovascular/cerebral dysfunction. With the present studies, we sought to determine the role of myeloid cells in the vascular vs. cerebral consequences of exposure to aircraft noise. Toxin-mediated ablation of lysozyme M^+^ (LysM^+^) myeloid cells was performed in LysMCre^iDTR^ mice carrying a cre-inducible diphtheria toxin receptor. In the last 4d of toxin treatment, the animals were exposed to noise at maximum and mean sound pressure levels of 85 and 72 dB(A), respectively. Flow cytometry analysis revealed accumulation of CD45^+^, CD11b^+^, F4/80^+^, and Ly6G^−^Ly6C^+^ cells in the aortas of noise-exposed mice, which was prevented by LysM^+^ cell ablation in the periphery, whereas brain infiltrates were even exacerbated upon ablation. Aircraft noise-induced increases in blood pressure and endothelial dysfunction of the aorta and retinal/mesenteric arterioles were almost completely normalized by ablation. Correspondingly, reactive oxygen species in the aorta, heart, and retinal/mesenteric vessels were attenuated in ablated noise-exposed mice, while microglial activation and abundance in the brain was greatly increased. Expression of phagocytic NADPH oxidase (NOX-2) and vascular cell adhesion molecule-1 (VCAM-1) mRNA in the aorta was reduced, while NFκB signaling appeared to be activated in the brain upon ablation. In sum, we show dissociation of cerebral and peripheral inflammatory reactions in response to aircraft noise after LysM^+^ cell ablation, wherein peripheral myeloid inflammatory cells represent a dominant part of the pathomechanism for noise stress-induced cardiovascular effects and their central nervous counterparts, microglia, as key mediators in stress responses.

## Introduction

According to the WHO Environmental Noise Guidelines for the European Region, traffic noise is an emerging cardiovascular risk factor [30] that has been linked to hypertension, diabetes, myocardial infarction, and stroke in epidemiological studies [41–43]. Recent translational research in mice demonstrated that aircraft noise induces vascular oxidative stress, vascular (endothelial) dysfunction and increased stress hormone levels, contributing significantly to the development of arterial hypertension and an increased presence of inflammatory cells such as leukocytes and macrophages in vascular tissue [34, 39, 59]. Noise also caused cerebral oxidative stress and inflammation, endothelial and neuronal nitric oxide synthase (e/nNOS) uncoupling, nNOS mRNA and protein down-regulation, and phagocytic NADPH oxidase (NOX-2) activation [34]. More recently, studies in humans demonstrated a correlation between road and aircraft noise exposure and subsequently, amygdala activation in the limbic system and vascular inflammation leading to an increase in major adverse cardiovascular events [53]. These novel neurobiological mechanisms may closely link noise to future cardiovascular disease and events [53].

The detrimental effects of noise pollution are exaggerated in the presence of established cardiovascular risk factors, as demonstrated in patients with coronary artery disease [43] and in translational studies in mice using the angiotensin-II model of hypertension [63]. This is related to increased reactive oxygen species (ROS) and inflammatory cytokine production triggering vascular recruitment and accumulation of immune cells causing endothelial dysfunction. Vascular tone depends on a complex interplay between the endothelium, vascular smooth muscle cells, immune cells, and inflammatory cytokines [67]. Noise exposure in mice leads to infiltration of CD45^+^, CD11b^+^, and Ly6g^+^Ly6c^+^ cells to the vascular wall [39] and a knockout of the phagocytic NOX-2 almost completely abolished the aircraft noise-induced vascular and cerebral oxidative stress and inflammation reaction and corrected endothelial dysfunction [34]. Accordingly, dysregulated inflammatory responses have been implicated as causal factors in noise-induced hypertension. Monocytes and macrophages accumulate in the aorta in response to angiotensin-II-induced arterial hypertension. When myeloid cells are depleted (using the LysMCre^iDTR^ model) [68] arterial hypertension development is blunted, oxidative stress and mRNA expression of pro-inflammatory cytokines are reduced, and aortic endothelial dysfunction is normalized. Therefore, with the present studies, we sought to investigate to what extent myeloid cell ablation may influence noise-induced peripheral vascular vs. cerebral inflammation and how this may impact vascular function using the LysMCre^iDTR^ model.

## Methods

### Animals

Animals were treated in accordance with the Guide for the Care and Use of Laboratory Animals as adopted by the U.S National Institutes of Health. Approval was granted by the Ethics Committee of the University Medical Center Mainz and the Landesuntersuchungsamt Rheinland-Pfalz (Koblenz, Germany; permit number: 23 177-07/G 15-1-094 and extensions). Male LysMCre^+/+^ iDTR^+/+^ were bred with C57BL/6J females purchased from Janvier according to a published protocol [32, 68]. The resulting pups were heterozygous LysMCre^+/−^ iDTR^+/−^, of which only the males were used in our experiments at an age of 6–8 weeks (female mice display inconsistent vascular responses due to hormonal changes during their menstrual cycle). For a minor part of the experiments (e.g., brain flow cytometry and vascular function measurement of mesenteric microvessels), homozygous LysMCre^+/+^ iDTR^+/+^ mice were used. A total number of 173 mice was used. All mice were housed in a standard 12-h light/dark cycle in an institutional animal facility with ad libitum access to standard rodent chow and water. After all treatments, the mice were sacrificed by cardiac puncture under isoflurane anesthesia and whole blood as well as organs were harvested for further investigations. Animal experiments were conducted at the same time of day with all four groups concurrently to account for circadian variation of several measured parameters.

### Administration of diphtheria toxin for LysM^+^ cell ablation

**Fig. 1:**
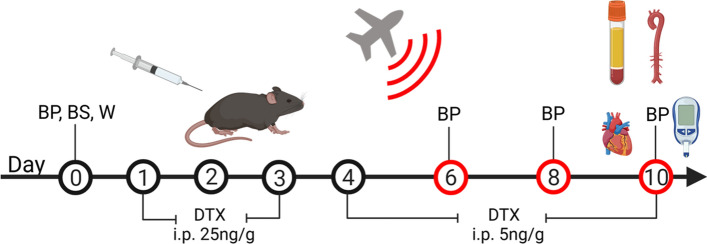
Scheme for ablation of LysM^+^ cells and noise exposure. Male LysMCre^+/−^ iDTR^+/−^ (LysMCre^iDTR^) mice were acclimatized to blood pressure measurement and baseline measurements were taken prior to treatment with diphtheria toxin. DTX was administered daily via i.p. injection for 10 days, first at a dose of 25 ng/g and then reduced to a dose of 5 ng/g. On day 6, after sufficient ablation, mice were exposed to aircraft noise for 4 days. Created with BioRender.com

Diphtheria toxin (Sigma; 322326) was dissolved in commercially available PBS to a concentration of 5 and 1 ng/µL. The toxin was then intraperitoneally injected once a day for 10 days, killing only the LysM^+^ cells expressing the inducible diphtheria toxin receptor, as previously reported [5]. For the first 3 days, mice were injected at a dose of 25 ng/g of mouse weight. For the remaining 7 days, the dose was reduced to 5 ng/g. This protocol was adapted from a published protocol [32, 68]. The time schedule of all treatments is shown in Fig. 1. The study protocol utilized four groups of age-matched male LysMCre^iDTR^ mice. Control (CTR, no noise exposure and no DTX treatment.), Noise (4 days of noise exposure), DTX (10 days of diphtheria toxin treatment), and DTX + Noise (10 days of DTX treatment and 4 days of noise exposure).

### Non-invasive blood pressure

Non-invasive blood pressure (NIBP) measurements were performed using a CODA High-Throughput 2 Noninvasive Blood Pressure System (Kent Scientific, Torrington, USA) according to a previously published protocol [34, 39, 63]. This method has been proofed in accuracy in comparison with radiotelemetric measurement of implanted catheter devices by Feng et al. [15]. Measurements were preceded by three training sessions to acclimate the animal before the baseline measurement and avoid stress reactions that may affect vascular tone and resting blood pressure. Conscious animals were allowed to enter the restraining tube freely, placed on a preheated platform at 32 °C, and allowed to rest for 15 min. The occlusion (O) cuff was fitted at the base of the tail and the volume–pressure recording (VPR) cuff was placed distal to the O-cuff. 15 NIBP measurements were taken per animal at each time point, the first five of which were discarded as acclimation cycles. The mean values of the remaining ten NIBP measurements were used in calculations. Measurements took place in a quiet setting at a set time each day to account for diurnal variation of blood pressure.

### Noise exposure

The noise chamber maintains the same housing conditions as the institutional animal facility to minimize transfer stress. Aircraft noise events are 43-s-long noise events, irregularly distributed over a 2-h sequence, which is repeated constantly over a 4-day period. The noise events are separated by irregularly distributed periods of silence to avoid early adaptation processes. Noise is applied through downward-facing speakers positioned approximately 30 cm above open mouse cages as described [34, 39]. The sound system is a Grundig MS 540 with a total output of 65 W. The noise events had a maximum sound pressure level of 85 dB(A) and a mean of 72 dB(A), volumes which are below the threshold for cochlear damage [65]. Sound pressure levels were calibrated with a Class II Sound level meter (Casella CEL-246) within one of the cages at initial setup.

### Isometric tension studies

After dissection, aortas were maintained in ice cold Krebs–Henseleit (KH) buffer and cleaned of perivascular fat. 4-mm segments of thoracic aortas were cut and mounted on force transducers within the organ bath chamber (25 mL). The rings were adjusted to a stable basal vascular tone and maximally constricted using bolus of 1 mL of 2 M KCl followed by a wash with KH buffer, then further boluses of 62.5, 125, 250, and 500 µL of 2M KCl to create a control contractile curve. The rings were then washed again and pre-constricted with prostaglandin F2α (PGF2α) to yield approximately 80% of the maximal tone induced by KCl bolus. Concentration–relaxation curves in response to increasing concentrations of acetylcholine (ACh) and nitroglycerin (NTG) were performed as previously described [40, 48]. Relaxation responses are reported as a percentage of maximal PGF2α constriction.

### Detection of oxidative stress via dihydroethidium staining

Dihydroethidium (DHE)-dependent fluorescence microtopography was used to determine the vascular ROS formation in cryo-sections of the aorta, as described [47, 49, 70]. Freshly harvested tissues were dissected and directly embedded in Tissue Tek (Sakura; 4538) and either immediately frozen in liquid nitrogen or incubated with the NOS inhibitor l-NAME (0.5 mM) before snap freezing. Samples were sliced on a cryostat (Leica; CM3050S). To perform the staining, slides were thawed and warmed to 38 °C and incubated with 1 µM DHE solution for 30 min. ROS-derived red fluorescence was detected using a Zeiss Axiovert 40 CFL microscope, Zeiss lenses, and Axiocam MRm camera. To investigate the involvement of eNOS uncoupling in ROS production and endothelial dysfunction, red fluorescence of DHE oxidation products was only quantified in the endothelial cell layer in aortic rings that were incubated with l-NAME. l-NAME increases endothelial cell-dependent ROS signal (DHE oxidation-dependent fluorescence) in control samples when eNOS is producing nitric oxide, and decreases endothelial cell-dependent ROS signal, when eNOS is producing superoxide (uncoupled enzymatic state) [11]. Of note, DHE fluorescence was quantified for a defined area in each aortic cryo-section and was normalized to this specific area to ensure comparable read-out for all evaluated animals. Normalization of the DHE signal to specific area was explained in detail for endothelial DHE staining in [11] and for vascular wall DHE staining in [50].

### Detection of oxidative stress via DHE-HPLC

Oxidative stress from superoxide was also measured by an HPLC-based method (modified from [77]) to quantify 2-hydroxyethidium levels as previously described [69, 70]. Briefly, tissue of aorta or heart was incubated with 50 µM DHE for 30 min at 37 °C in PBS. Tissues were homogenized in 50% acetonitrile/50% PBS using a glass homogenizer (heart) or pulverized in a mortar under liquid nitrogen and resuspended in homogenization buffer (aorta), centrifuged and 50 µL of the supernatant were subjected to HPLC analysis. The system consists of a control unit, two pumps, mixer, detectors, column oven, degasser and an autosampler (AS-2057 plus) from Jasco (Groß-Umstadt, Germany), and a C18-Nucleosil 100-3 (125 × 4) column from Macherey & Nagel (Düren, Germany). A high pressure gradient was employed with acetonitrile and 50 mM citrate buffer (pH 2.2) as mobile phases with the following percentages of the organic solvent: 0 min, 36%; 7 min, 40%; 8–12 min, 95%; 13 min, 36%. The flow was 1 mL/min and DHE was detected by its absorption at 355 nm, whereas 2-hydroxyethidium was detected by fluorescence (Ex. 480 nm/Em. 580 nm).

### Western blot analysis of protein expression

Protein was isolated from frozen tissue by homogenization under nitrogen, then incubated in lysis buffer for 30 min with intermittent vortexing. Following incubation, the samples were centrifuged at 12,000 × *g* at 4° for 20 min. The resulting protein-rich lysate was extracted from the pellet and measured in concentration by Bradford assay. Samples were then prepared in Laemmli buffer to a concentration of 2 µg/µL. Gel electrophoresis was conducted with 30 µg of protein on 6 and 12% gels, transferred onto a nitrocellulose membrane, and blocked with 5% milk protein in TBS-T. Primary antibodies NLRP3 (Cell Signaling #15101, 1:750) and TXNIP (Cell Signaling #14715, 1:1000) were incubated overnight at 4° with either α-actinin or β-actin as loading controls (Sigma, 1:2500). Membranes were then washed 3 × in TBS-T and incubated with respective anti-mouse or anti-rabbit peroxidase-coupled secondary antibodies (Vektor PI-2000 or PI-1000, 1:10,000) for 2 h at room temperature, at which point positive bands were detected using ECL development (Thermo Fisher, 32106) and Chemilux Imager (CsX-1400M, Intas). Densitometric quantification was performed using ImageJ software [42].

### Dot blot analysis of protein modification and expression

Following protein isolation and determination of concentration as described in the Western blot section, 20 µg of protein was transferred into each well to deposit on the nitrocellulose membrane (Sigma Aldrich, WHA10402506) via a Minifold I vacuum Dot-Blot system (Schleicher&Schuell, 10484138CP) [34, 39], washed twice with 200 µL of PBS then dried for 60 min at 60 °C to adhere the proteins. The membranes were then cut and incubated in Ponceau S solution (Sigma, P7170) for protein visualization. The stain was removed and the membrane blocked for 1 h at room temperature with 5% milk in PBS-T. The membranes were incubated with antibodies against malondialdehyde (MDA)-positive proteins (Abcam, ab27642, 1:1000) as well as CD68 (Abcam, ab955, 1:1000), and interleukin 6 (Abcam, ab6672, 1:1000) overnight at 4 °C. The membranes were then treated in the same manner as those of Western blots.

### Immunohistochemistry of aortic rings

Procedure for immunohistochemistry has been previously described [34, 39, 63]. Briefly, aortic segments retaining adventitial and perivascular tissue were fixed in 4% formaldehyde, embedded in paraffin, and sliced into sections of 5 µm. Following deparaffinization, samples were blocked with normal horse blocking solution (Vector) and stained with a primary antibody against NOX-2 (1:200, #LS-B12365, LS Bio) or 3-nitrotyrosine (1:200, Merck-Millipore, Darmstadt, Germany), biotinylated with a secondary antibody (Thermo Fisher Scientific, Waltham, MA), and then imaged with Image ProPlus 7.0 (Media Cybernetics, Rockville, MD).

### Quantitative reverse transcription real-time PCR (qRT-PCR)

Total mRNA from aortic and brain tissue was isolated using the acid guanidinium thiocyanate–phenol–chloroform extraction method. 125 ng of total RNA was used for quantitative reverse transcription real-time PCR (qRT-PCR) analysis using QuantiTect Probe RT-PCR kit (Qiagen) as described previously [22, 51]. Primer–probe–mixes purchased from Applied Biosystems (Foster City, CA) were used to analyze the mRNA expression patterns of NADPH oxidase 2 (*NOX-2 Mm00432775_m1*), eNOS (*NOS3, Mm00435204_m1*) and vascular cell adhesion protein 1 (*VCAM-1, Mm00449197_m1*) in aorta and NFκB2 (*NFκB2, Mm00479810_g1*), CD40L (*CD40L, Mm_00441911_m1*) in brain. All samples and tissues were normalized on the *TATA box binding protein* (*TBP*, Mm_00446973_m1) as an internal control. For quantification of the relative mRNA expression, the comparative ΔΔCt method was used. Expression of target gene in each sample was expressed as the percentage of unexposed wild type.

### ELISA for catecholamines and corticosterone

Circulating catecholamines (adrenaline and noradrenaline) were determined in mouse plasma using a commercial enzyme-linked immunosorbent assay (ELISA) kit (TriCat, IBL, RE59395) following the instructions of the vendor [39]. Corticosterone plasma levels were determined by commercial ELISA (Enzo, ADI-900-097) [39].

### Immunohistochemistry for brain microglia

Immunohistochemistry for microglia was performed on 4–5-μm-thick de-paraffinized sagittal brain sections as described previously [34]. Endogenous peroxidase was blocked using H_2_O_2_ (3%) in PBS-T for 30 min. Subsequently, sections were washed twice with 1 mL PBS-T (2 min) followed by incubation in 5% NGS for 20 min at RT, followed by 2 × 2 min washing with PBS-T. Blocking of endogenous biotin was performed with an Avidin/Biotin Blocking Kit (SP-2001; Vector laboratories). Sections were then incubated with the respective primary antibody overnight at 4 °C (polyclonal rabbit anti-Iba1, 1:400, WAKO). After washing with PBS-T (2 × 3 min), immunoreactivity was visualized by the avidin‐biotin complex method and sections were developed in diaminobenzidine (DAB, Sigma). For negative controls, the primary antibodies were omitted. The micrographs were processed using ImageJ software (https://imagej.net/ImageJ [60]) as follows. First, a number of microglia were counted using the cell counter plugin and calculated as number of microglia/mm^2^. Then, color deconvolution function (vector H DAB) was used to unmix RGB images. Micrographs from channel two (brown signal) were then processed using the threshold function. The Iba-1 immunoreactive area in % was then quantified using the analysis function.

### Flow cytometry of aortic tissue lysates as well as whole blood

Flow cytometry of aortas and whole blood was performed as described previously [33, 68]. Briefly, cleaned aortic vessels were digested with liberase™ (1 mg/mL, Roche, Basel, Switzerland) for 30 min at 37 °C and passed through a cell strainer (70 μm) to yield a single-cell suspension. Single-cell suspensions were treated with Fc-block (anti-CD16/CD32), washed, and surface stained with CD45 APC-efluor 780 (#47-0451-82, 2 µg/mL), NK1.1 PE-Cy7 (#25-5941-81, 2 µg/mL), F4/80 APC (#17-4801-82, 4 µg/mL) from eBioscience (San Diego, CA) and TCR-β V450 (#560706, 2 µg/mL), CD11b PE (#553311, 2 µg/mL), Ly6G FITC (#551460, 5 µg/mL), and Ly6C PerCP-Cy.5.5 (#560525, 2 µg/mL) from BD Biosciences (San Diego, CA). Dead cells were excluded by staining with Fixable Viability Dye eFluor506 (#65–0866-14, 1:1000, eBioscience, San Diego, CA). Based on a live gate, events were acquired and analyzed using a BD FACS CANTO II flow cytometer (Becton Dickinson, Franklin Lakes, NJ) and FACSDiva software (Becton Dickinson, Franklin Lakes, NJ), respectively. Gating was performed according to a previously published strategy [63].

### Flow cytometry of brain tissue lysates

The protocol was previously published [21, 57]. Brain tissue was dissected from LysMCre^+/+^iDTR^+/+^ mice which were transcardially perfused with 0.9% NaCl solution (B. Braun, Melsungen, Germany). The dissected brains were digested with papain (1 mg/mL, Sigma-Aldrich, St. Louis, MO) and DNase I (100 μg/mL, Roche, Basel, Switzerland) for 30 min at 37 °C and were mechanically homogenized using the gentleMACS™ Dissociator (Miltenyi, Bergisch Gladbach, Germany) during the incubation time. The cell suspension was filtered through a cell strainer (70 μm) to yield a single-cell suspension. Cells were separated using a 30%:70% Percoll (Sigma-Aldrich, St. Louis, MO) gradient centrifugation for 45 min, 500 ×*g* at 18 °C without brakes. Myelin was discarded, and the cells at the interface were collected and washed in PBS containing 2% FCS (Thermo Fisher, Waltham, MA). Single-cell suspensions were treated with Fc-block (anti-CD16/CD32, BioXCell, Lebanon, NH), washed and surface-stained with CD45 BV510 (#103138, BioLegend, San Diego, CA, 0.7 µg/mL), CD11b PE-Cy7 (#101216, BioLegend, San Diego, CA, 0.2 µg/mL), CD80 PerCP-Cy™5.5 (#560526, BD Biosciences, Franklin Lakes, NJ, 0.5 µg/mL), CD86 FITC (#553691, BD Bioscience, Franklin Lakes, NJ, 0.5 µg/mL), MHC Class II eFl450 (#48-5321-82, eBioscience, San Diego, CA, 0.05 µg/mL), Ly6G PE (#127608, BioLegend, San Diego, CA, 0.4 µg/mL), Ly6C V450 (#560594, BD Bioscience, Franklin Lakes, NJ, 0.7 µg/mL), and F4/80 APC (#123116, BioLegend, San Diego, CA, 1 µg/mL) for 30 min at 4 °C. Dead cells were excluded by staining with Fixable Viability Dye eFluor™ 780 (#65-0865-18, 1:1000, eBioscience, San Diego, CA). For intracellular staining, cells were fixed and permeabilized using the Foxp3/Transcription Factor Staining Buffer Set (#00-5523-00, eBioscience, San Diego, CA) and stained for 12 h at 4 °C with CD68 APC (#137008, BioLegend, San Diego, CA, 0.4 µg/mL). The samples were acquired and analyzed using a BD FACS CANTO II flow cytometer (Becton Dickinson, Franklin Lakes, NJ) and FACSDiva software (Becton Dickinson, Franklin Lakes, NJ). Microglia were identified by gating on live, single, CD45^intermediate^ CD11b^+^ cells. Brain infiltrates were separated according to their expression levels of CD11b into CD45^high^ CD11b^+^ and CD45^high^ CD11b^-^ populations.

### Microvascular reactivity and oxidative stress

Microvascular reactivity was measured in first-order arterioles of isolated retinas using video microscopy as previously described [16, 18]. After dissection, eyes were transferred into ice cold KH buffer for preparation of the ophthalmic artery, isolation of the retina, cannulation the ophthalmic artery and placing the retina onto a transparent plastic platform. Subsequently, retinal arterioles were pressurized to 50 mmHg, visualized under brightfield conditions, and equilibrated for 30 min. Mesenteric small arteries/arterioles of the 6th and 7th order were isolated and measured as previously described for other small blood vessels with minor modifications [19, 74]. Briefly, a mesenteric vascular tree was isolated and cleaned from surrounding tissue by fine-point tweezers and Vannas scissors. Next, a micropipette was inserted into the lumen of the proximal part of the vascular tree (typically into the 4th branch) and advanced into the 6th or 7th branch using the lumen as a guide channel. Once the micropipette tip was placed, the vessel was tied to the pipette with 10.0 nylon suture material. The other end of the arteriole was tied to another micropipette. The vessel was then pressurized to 50 mmHg and visualized under an inverted microscope. Next, concentration–response curves for the thromboxane mimetic, U46619 (10^−11^ to 10^−6^ M; Cayman Chemical, Ann Arbor, MI, USA), were conducted for both retinal arterioles and mesenteric small arteries/arterioles. For measurement of vasodilation responses, vessels were then preconstricted to 50–70% of the initial luminal diameter by titration of U46619 and responses to the endothelium-dependent vasodilator, acetylcholine (10^−9^ to 10^−4^ M; Sigma-Aldrich, Taufkirchen, Germany) and to the endothelium-independent nitric oxide donor, sodium nitroprusside (SNP, 10^−9^ to 10^−4^ M, Sigma-Aldrich), were determined. ROS formation was measured in retinal blood vessels in 10 µm cryosections of the retina by dihydroethidium (DHE, 1 µM)-derived fluorescence (518/605 nm excitation/emission), as previously described [17, 75].

### Statistical analysis

All results are expressed as mean ± SEM and calculated with GraphPad Prism 8.01. Two-way ANOVA with Bonferroni’s correction were used for comparisons of concentration–relaxation curves and blood pressure over time. For endpoint blood pressure, rtPCR, flow cytometry, western blot, dot blot and oxidative stress parameters, one-way ANOVA with Bonferroni’s or Tukey’s correction was used for the comparison of multiple means. All *p* < 0.05 are considered significant and denoted by: * vs. Control, # vs. Noise, $ vs. DTX, and + vs. DTX + Noise.

## Results

### Protective effects of LysM^+^ cell ablation on blood pressure elevation and impairment of endothelial function by noise exposure

**Fig. 2:**
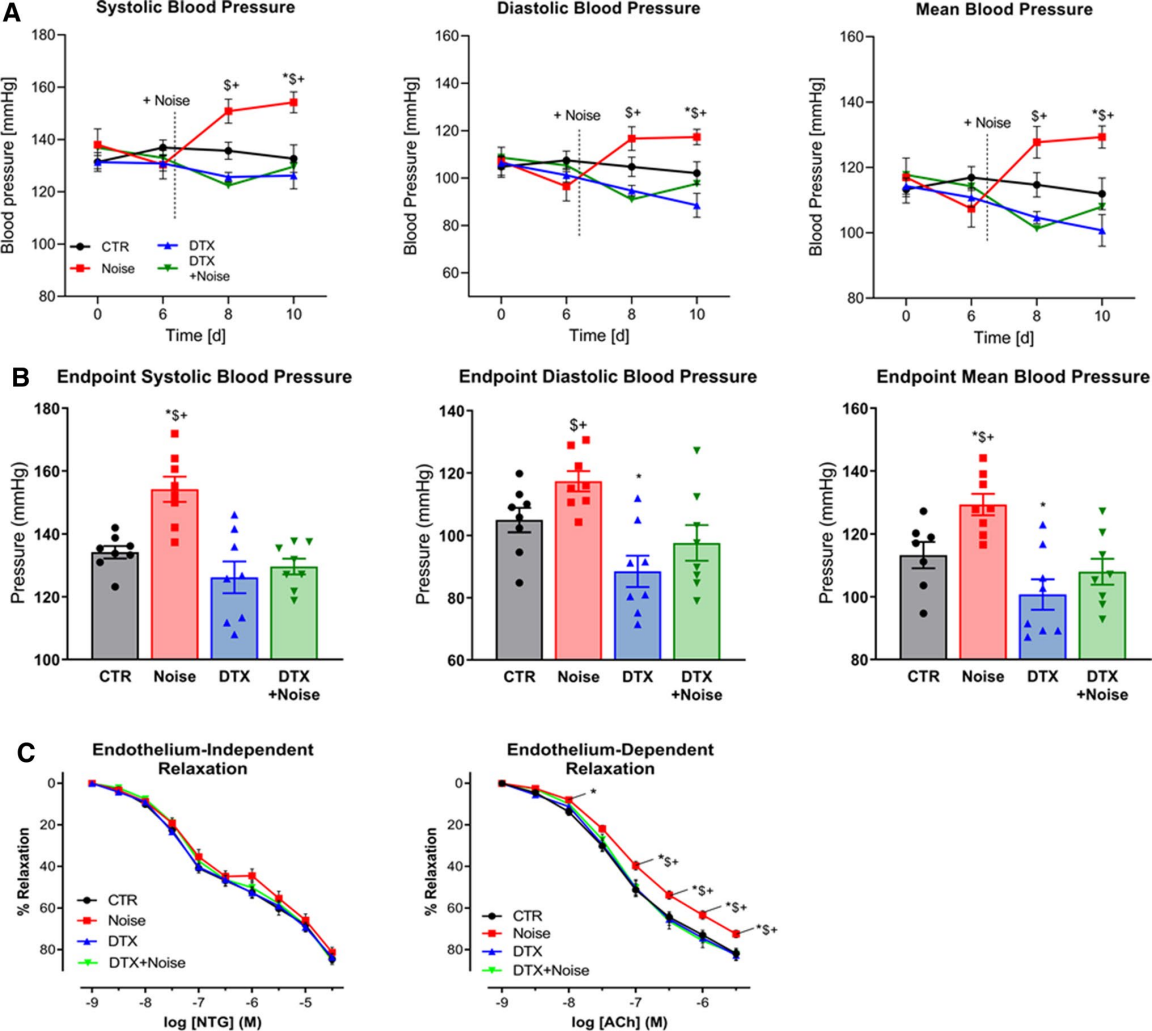
Effects of ablation of LysM^+^ cells on blood pressure and endothelial function of aircraft noise-exposed animals. **a** Aircraft noise increased systolic, diastolic, and mean blood pressure, all of which was prevented by treatment with DTX. Noise was applied on day 6. **b** Systolic, diastolic, and mean blood pressure on the final day of the exposure regimen. Ablation of LysM^+^ cells completely abolished noise-induced increases in blood pressure. **c** Noise caused a significant degree of endothelial dysfunction (ACh-response) that was completely prevented by ablation therapy, whereas endothelium-independent relaxation (NTG-response) was not changed in any group. Data points are measurements from individual animals (**a**, **b**) and data in (**c**) are the mean of *n* = 19–27 independent measurements; two-way ANOVA with Bonferroni’s multiple comparison test (**a**, **c**) or one-way ANOVA with Tukey’s multiple comparison test (**b**). ^*^P < 0.05 vs. Control; ^$^*P* < 0.05 vs. DTX, ^+^*P* < 0.05 vs. DTX + Noise

Systolic and diastolic blood pressure were significantly increased by noise exposure at days 8 and 10 of the treatment regimen (days 2 and 4 after beginning of noise exposure), respectively. Ablation of LysM^+^ cells had only minor effects on blood pressure in mice without noise exposure, but prevented a blood pressure increase in the noise-exposed animals (Fig. 2a). The endpoint blood pressure values (day 10 of treatment regimen) showed a significant difference in blood pressure between noise and all other groups (Fig. 2b). The acetylcholine (ACh) dose–response relationship was significantly impaired in response to noise, while endothelium-independent relaxation to nitroglycerin was not impaired (Fig. 2c). LysM^+^ cell ablation did not modify ACh-responses of mice aorta unexposed to noise, but prevented endothelial dysfunction in the noise-exposed group. Aortas of mice from the DTX + Noise group showed identical endothelium-dependent and -independent vasodilation as compared to the control group (Fig. 2c).

### Prevention of ROS formation in vascular and cardiac tissue

**Fig. 3:**
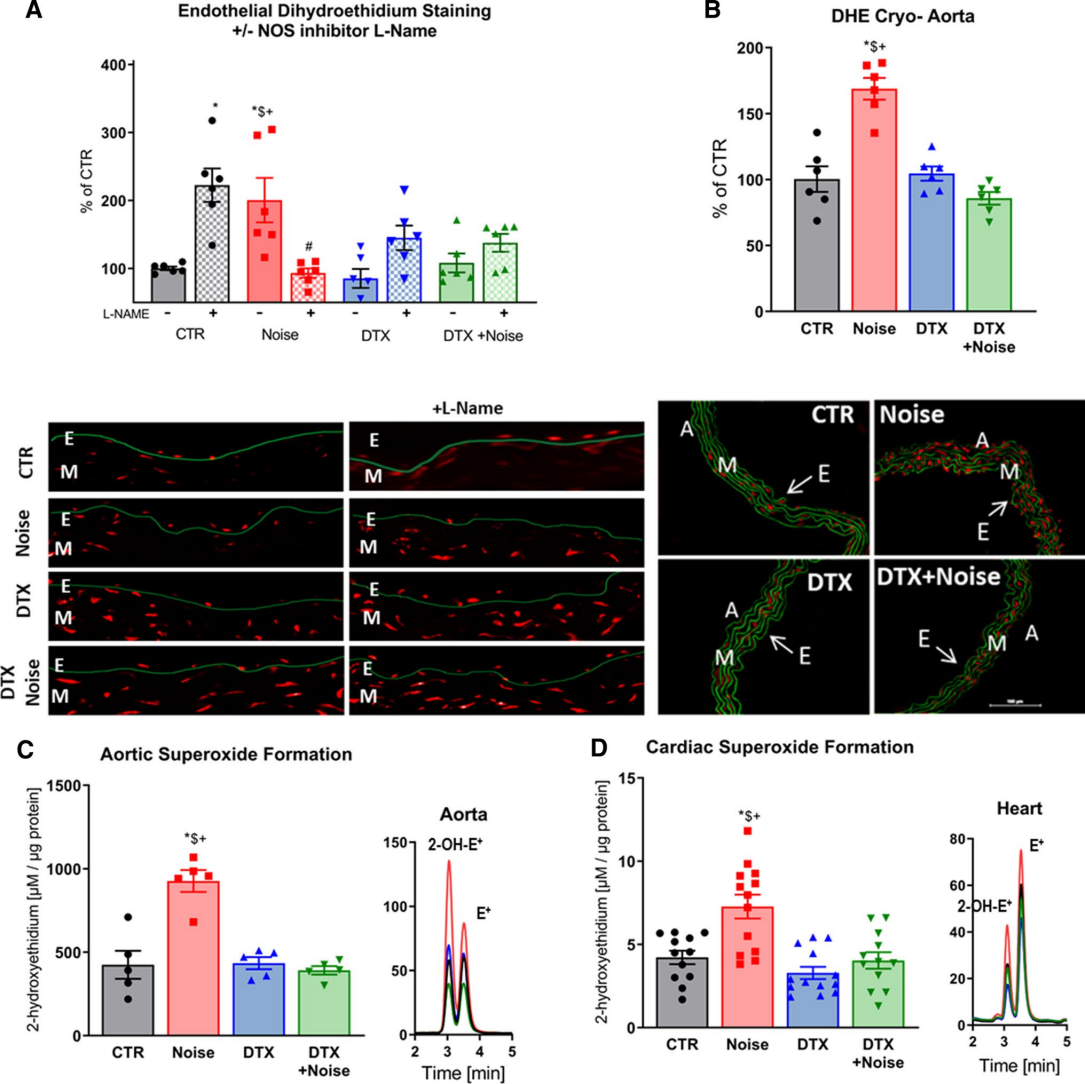
Noise-induced increases of eNOS uncoupling as well as vascular and cardiac oxidative stress are successfully corrected by ablation of LysM^+^ cells. **a** In control vessels, the eNOS inhibitor l-NAME increased vascular superoxide levels while decreasing it in noise-exposed animals, findings compatible with eNOS uncoupling. In contrast, after ablation of LysM^+^ cells, l-NAME increased the vascular DHE signal indicating that eNOS uncoupling was prevented. **b** DHE staining of aortic cryo-sections revealed an increase in ROS production to noise that was prevented by ablation. Red fluorescence indicates the signal corresponding to oxidation product and green indicates the autofluorescence of aortic laminae. The scale bar reflects 100 µm. **c**, **d** Likewise, quantitative HPLC analysis of the superoxide-specific DHE product, 2-hydroxyethidium, established increases in response to noise in the aorta and heart that were normalized by ablation. Representative chromatograms are shown besides the quantification. Data points are measurements from individual animals; one-way ANOVA with Tukey’s multiple comparison test (**a**–**d**). ^*^P < 0.05 vs. Control; ^$^*P* < 0.05 vs. DTX, ^+^*P* < 0.05 vs. DTX + Noise

Noise exposure caused uncoupling of eNOS, exhibited by an increase in DHE signal in the endothelial cell layer and significant suppression by l-NAME (Fig. 3a). Endothelial eNOS uncoupling was mostly prevented by LysM^+^ cell ablation. Prevention of eNOS uncoupling was also evident in both LysM^+^ cell ablation groups by an increase in DHE signal upon treatment with l-NAME. DHE staining in aortic cryo-sections revealed that noise increases vascular superoxide production, which is prevented by ablation of LysM^+^ cells (Fig. 3b). Likewise, using quantitative HPLC analysis of the superoxide-specific DHE product, 2-hydroxyethidium, we established that noise increased superoxide production in aortic and cardiac tissue compared to control, which was prevented by DTX treatment (Fig. 3c, d).

### Prevention of leukocyte extravasation into the aortic endothelium

**Fig. 4:**
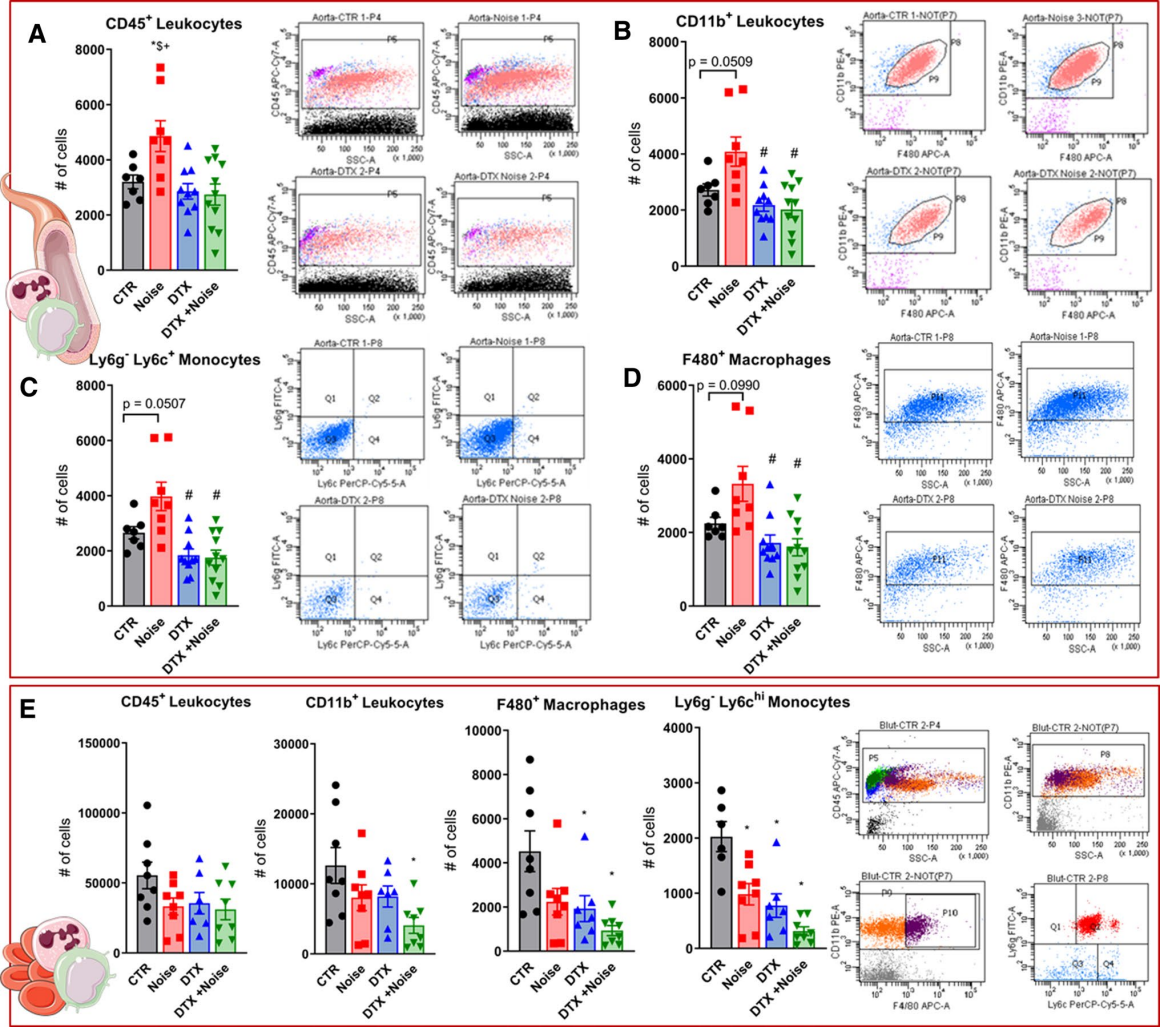
Flow cytometry of aortic lysates and whole blood demonstrates leukocyte extravasation into the aortic endothelium by noise and prevention by LysM^+^ cell ablation. Flow cytometry and representative plots in aortic lysates show an increase in count following noise exposure and a reduction in count in all leukocytes (**a**) as well as innate immune leukocytes (**b**). Specifically, monocytes (**c**) and macrophages (**d**) were reduced below baseline control counts and no increase was found in the aorta following both noise exposure and LysM^+^ cell ablation. **e** All immune cell subsets were found decreased in whole blood of noise-exposed CTR mice or in the DTX-treated groups. Data points are measurements from individual animals; one-way ANOVA with Tukey’s multiple comparison test (**a**–**e**). ^*^P < 0.05 vs. Control; ^#^*P* < 0.05 vs. Noise; ^$^*P* < 0.05 vs. DTX, ^+^*P* < 0.05 vs. DTX + Noise

Flow cytometry analysis showed an increase in the presence of all leukocytes (CD45^+^), myeloid cells (CD11b^+^), macrophages (F4/80^+^), and monocytes (Ly6G^−^Ly6C^+^) in the aortas of mice subjected to 4 days of noise exposure. Depletion of LysM^+^ cells prevented this increase (Fig. 4a–d). Complementary to the influx of immune cells into the aorta, we observed a drop in all subsets (CD45^+^, CD11b^+^, Ly6G^−^Ly6C^+^, F4/80^+^) in whole blood of noise-exposed mice (Fig. 4e) that we interpret as a shift of these cells from the blood stream to the aortic tissue and potentially other organs.

### Effects in mRNA expression and oxidative stress markers following noise exposure

**Fig. 5:**
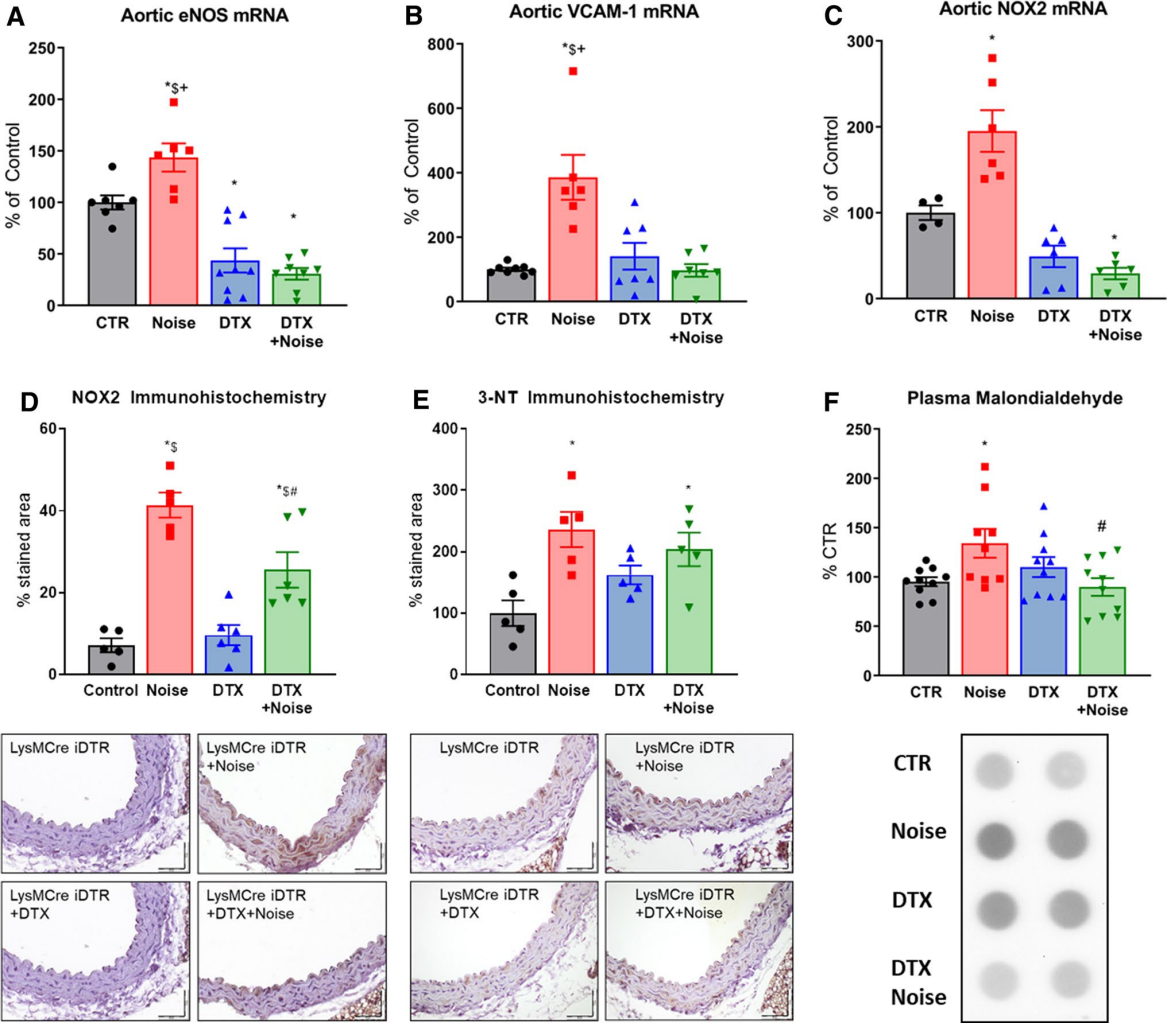
Ablation protects from noise-induced increases in mRNA expression of endothelial nitric oxide synthase (eNOS), oxidative stress and inflammatory parameters and plasma oxidative stress markers. Noise increased mRNA expression of *eNOS, VCAM-1, NOX-2* expression (**a**–**c**). Ablation of LysM^+^ cells reduced *eNOS* and *NOX-2* mRNA (**a**, **c**) below control levels, while the expression of *VCAM-1* (**b**) remained at baseline. **d**, **e** Protein expression of NOX-2 and abundance of 3-nitrotyrosine (3-NT)-positive proteins in the aorta as determined by immunohistochemistry were increased by noise and partially normalized by DTX treatment. Representative immunohistochemical images are shown below the quantification and the scale bars reflect 50 µm. **f** Oxidative stress marker, malondialdehyde assessed by dot blot analysis, was increased by noise and normalized by LysM^+^ cell ablation. Data points are measurements from pools of 3–4 aortas (**a**–**c**), number of animals (**d**, **e**) or pools of plasma from 2–4 animals per data point (**f**); one-way ANOVA with Tukey’s multiple comparison test (**a**–**f**). ^*^P < 0.05 vs. Control; ^#^*P* < 0.05 vs. Noise; ^$^*P* < 0.05 vs. DTX, ^+^*P* < 0.05 vs. DTX + Noise

Noise induced a significant increase in expression of *eNOS, VCAM-1*, and *NOX-2* mRNA as well as NOX-2 protein as previously reported [28]. While there was a reduced expression for *eNOS* and *NOX-2* in DTX-treated LysMCre^iDTR^ mice, there were no increases in expression in LysM^+^ cell-depleted mice in response to noise exposure (Fig. 5a–c). A similar observation was made for NOX-2 protein expression in the aorta that was increased by noise, which was prevented at least in part by DTX treatment (Fig. 5d). In line with the upregulation of NOX-2 expression, the markers of oxidative stress, 3-nitrotyrosine, and malondialdehyde were increased in the aorta as well as plasma of noise-exposed control mice, whereas no such increase was observed upon noise exposure of LysM^+^ cell ablated mice (Fig. 5e, f).

### Normalization of endothelium-dependent vasodilation and ROS formation in retinal arterioles and mesenteric small arteries/arterioles

**Fig. 6:**
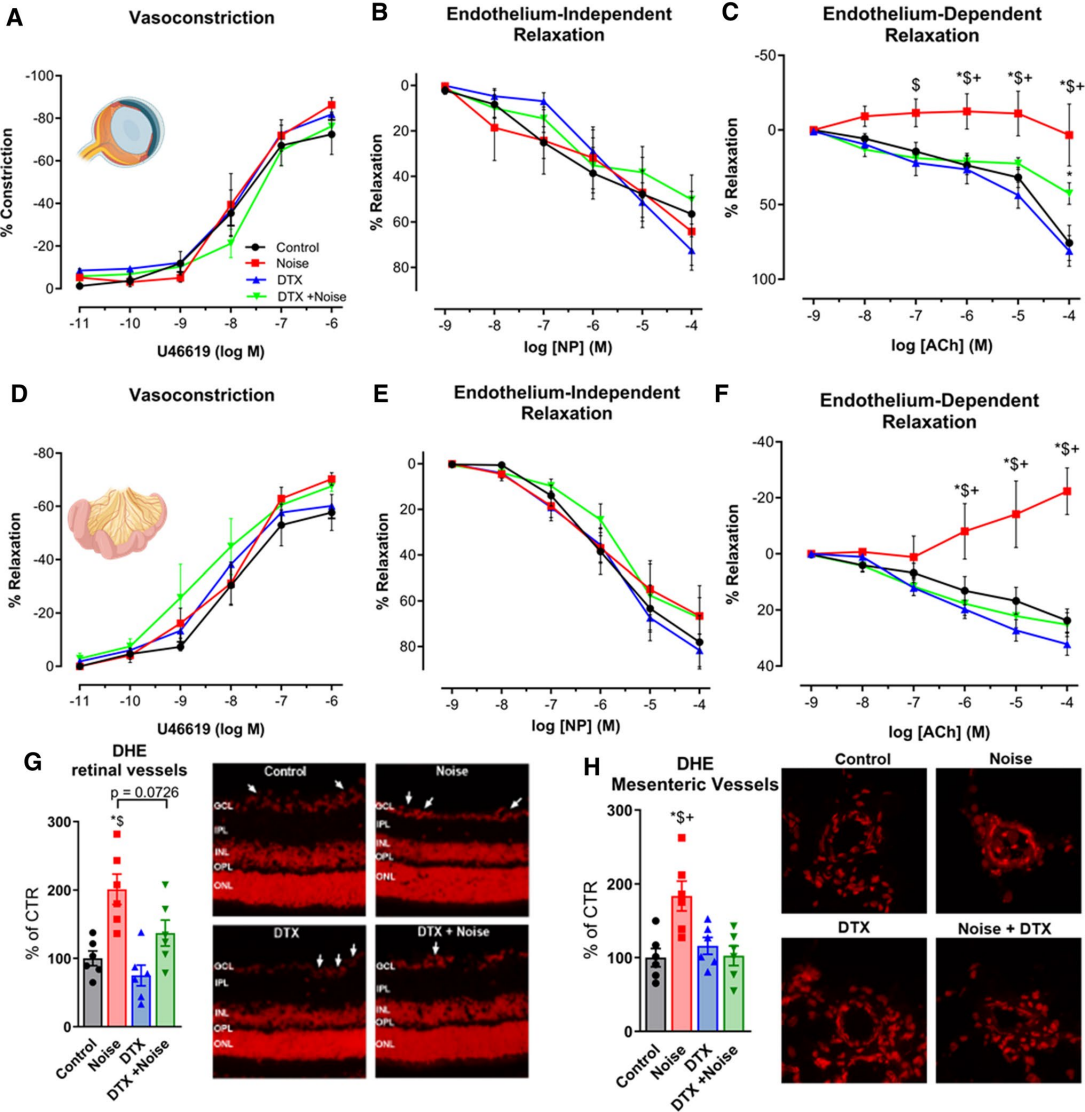
Noise-induced impairment of vasodilation and increase of oxidative stress in the retinal and mesenteric microvasculature is mostly prevented by ablation LysM^+^ cells. (**a**–**c** and **d**–**f**) In retinal and mesenteric arterioles, noise caused a marked degree of endothelial dysfunction (impaired ACh-response), while responses to the endothelium independent vasodilator nitroprusside (NP) and to the vasoconstrictor U46619 (thromboxane A2 agonist) remained unchanged. Ablation of LysM^+^ cells partially normalized the ACh-dose response relationship in retinal vessels and completely normalized it in mesenteric vessels. (**g**, **h**) DHE staining revealed an increase in ROS production in retinal and mesenteric vessels upon exposure to noise, with no effect by DTX treatment alone and partial or total prevention of the increase by DTX treatment prior to noise. Representative images of DHE-stained retinal and mesenteric cryosections are shown besides the densitometric quantification. The white arrows point to retinal vascular cross-sections. *GCL* ganglion cell layer; *IPL* inner plexiform layer; *INL* inner nuclear layer; *OPL* outer plexiform layer; *ONL* outer nuclear layer. Data points are measurements from individual animals (**g**, **h**) or *n* = 6 for (**a**–**c**) or *n* = 3–4 for (**d**–**f**); one-way ANOVA with Tukey’s multiple comparison test (**a**–**c**). two-way ANOVA with Bonferroni’s multiple comparison test (**d**–**f**). ^*^P < 0.05 vs. Control; ^#^*P* < 0.05 vs. Noise; ^$^*P* < 0.05 vs. DTX, ^+^*P* < 0.05 vs. DTX + Noise

The smooth-muscle-dependent vasoconstrictor, U46619, was used to generate constriction of retinal arterioles, demonstrating no difference between any of the groups (Fig. 6a). Similarly, there was no detectable difference from control in smooth-muscle dependent vasodilation via nitroprusside in any of the groups (Fig. 6b). In contrast, endothelium-dependent relaxation induced by acetylcholine illustrated a significant reduction in vasodilation in noise-exposed mice, which was partly alleviated by LysM^+^ cell ablation (Fig. 6c). DHE staining in retinal vessels revealed a similar pattern, with a significant increase in DHE signal upon noise exposure, where ablation generally defended against ROS formation (Fig. 6g). In mesenteric vessels, no significant differences were found in the U46619-induced constriction in any of the groups in homozygous males (Fig. 6d) nor in the endothelium-independent vasodilation (Fig. 6e). A stark contrast was found in endothelium-dependent vasodilation between the noise-exposed group and all others, demonstrating clear protection in the DTX + Noise group (Fig. 6f), whose function remained similar to control. These responses were mirrored in heightened levels of ROS formation upon noise exposure and normalization of ROS formation in DTX + Noise (Fig. 6h).

### Increased microglial presence, neuroinflammatory phenotype, and neurohormonal stress response

**Fig. 7:**
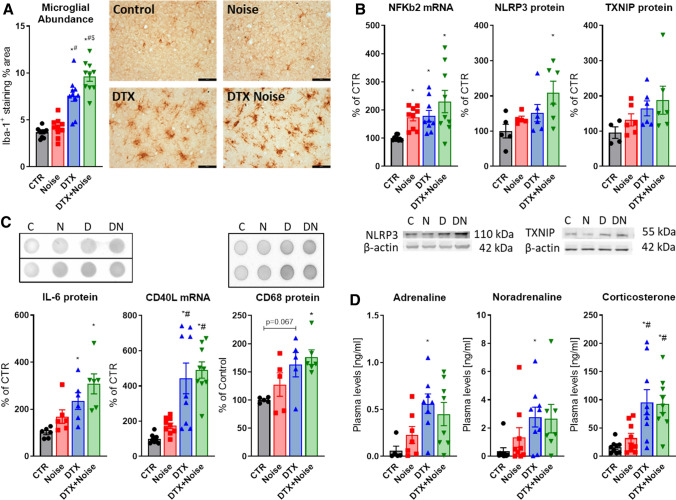
Diphtheria toxin treatment of LysMCre^iDTR^ mice fails to prevent neuroinflammation in the brain and stress responses by noise. **a** Iba-1 staining revealed no ablation of microglia in LysMCre^iDTR^ mice. Interestingly, Iba-1^+^ cells and % of Iba-1^+^ area were even increased in both DTX-treated groups and further aggravated by noise exposure. Representative immunohistochemical images are shown besides the quantification and the scale bar reflects 50 µm. **b** Presence of a neuroinflammatory phenotype was supported by higher levels of NFkB mRNA as well as NLRP3 and TXNIP protein expression in brains of LysMCre^iDTR^ mice with DTX treatment, which was exacerbated by noise exposure. Representative western blot images are shown below the densitometric quantification. **c** IL-6 and CD68 protein as well as CD40L mRNA expression were slightly increased by noise and further exacerbated in the DTX groups. Representative dot blot images are shown above the densitometric quantification. **d** Neuronal stress response and release of stress hormones adrenaline, noradrenaline and corticosterone were also higher in the LysMCre^iDTR^ mice with DTX treatment. Data points are measurements from individual animals; one-way ANOVA with Tukey’s multiple comparison test or respective non-parametric test (**a**–**d**). ^*^P < 0.05 vs. Control; ^#^*P* < 0.05 vs. Noise; ^$^*P* < 0.05 vs. DTX

The moderate inflammation induced by noise was increased following LysM^+^ cell ablation. An increase in Iba-1^+^ staining in the DTX-treated groups and altered microglial phenotype indicates an increased presence and potentially activation of microglia (Fig. 7a, perimeter evaluation not shown). The immunohistochemical data were also supported by increased levels of cerebral NFκB subunit 2 mRNA as well as NLRP3 and TXNIP protein (Fig. 7b). Downstream of the NFκB-NLRP3-TXNIP signaling axis, IL-6 protein, CD40L mRNA as well as CD68 protein were upregulated by trend by noise and further increased in the DTX-treated groups supporting the neuroinflammatory phenotype (Fig. 7c). The stress response and release of stress hormones adrenaline, noradrenaline, and corticosterone was also more pronounced in the LysMCre^iDTR^ mice with DTX treatment (Fig. 7d). In line with the neuroinflammatory phenotype in the DTX groups, we also observed a trend of elevated cerebral malondialdehyde and p67^phox^ levels, a marker of lipid peroxidation and a regulatory subunit of the NOX-2, in all DTX-treated mice, and this tendency was most pronounced for malondialdehyde and even became significant for p67^phox^ in the noise-exposed DTX mice (not shown). Cerebral eNOS protein expression showed an almost identical pattern with most pronounced increase in the noise-exposed DTX-treated mice (not shown). Noise exposure increased all these parameters in the control mice at least by trend.

### Evidence of microglial activation and peripheral infiltration in the brain

**Fig. 8:**
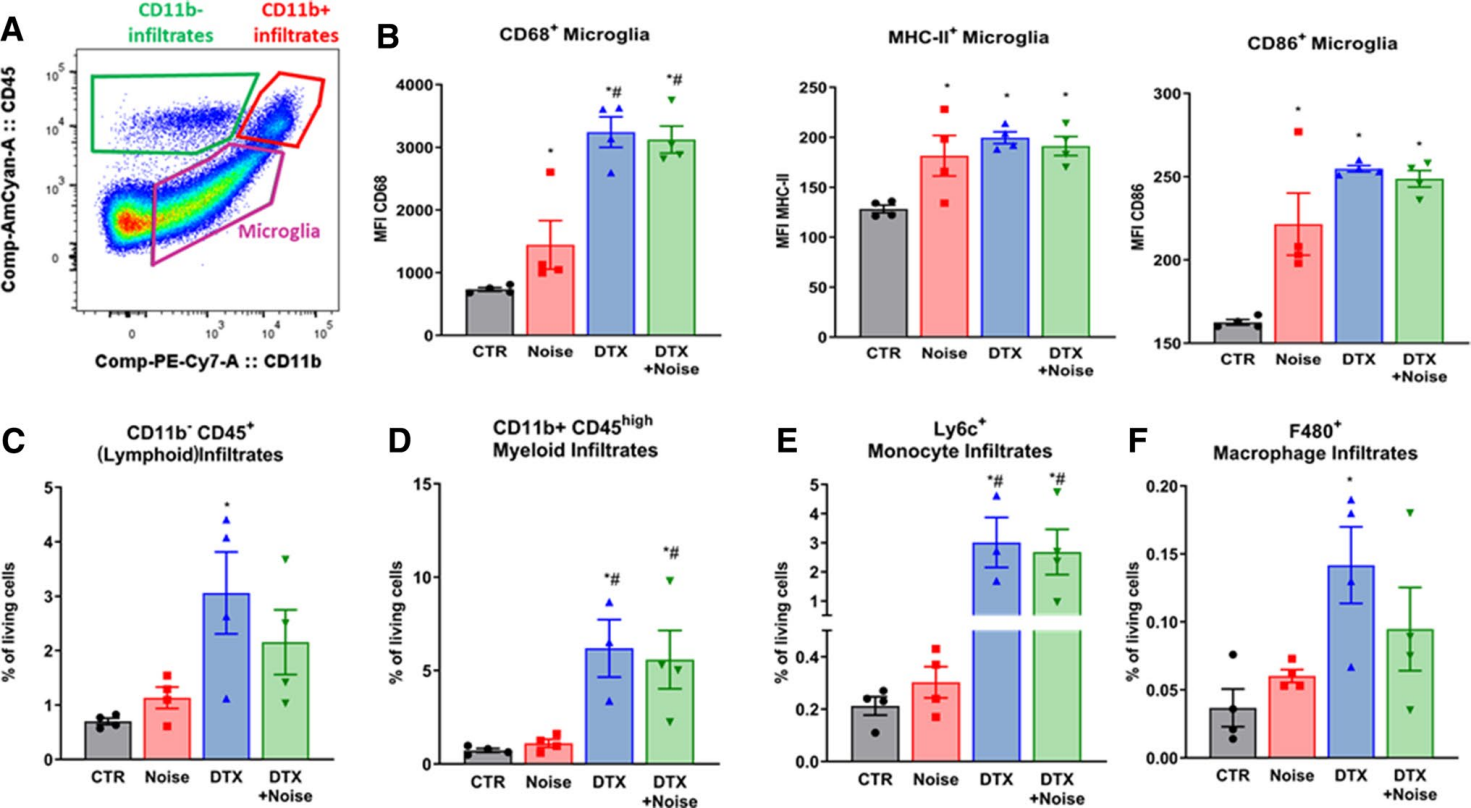
Both noise and DTX treatment induce microglial activation and DTX also causes peripheral immune infiltration into the brain. **a** Microglia are moderate expressors of both CD11b and CD45, whereas peripheral immune cells express high levels of CD45. Using flow cytometry with this gating strategy, we were able to evaluate the activation of microglia and identify CD11b^+^ and CD11b^−^ infiltrates from the periphery in the brains of LysMCre^+/+^iDTR^+/+^ mice. **b** CD68, CD86, and MHC-II are markers of microglial activation, demonstrating that both noise and DTX activate central immune responses. **c** CD45^hi^ CD11b^−^ (e.g., lymphoid) cells showed increased infiltration into the brain upon DTX treatment. **d** Myeloid infiltrates were detected by their expression of CD11b and their high expression of CD45. Despite ablation of these cells in the periphery, there was considerable migration of myeloid cells into the brain following DTX treatment, which also comprised monocyte- (**e**) and macrophage- (**f**) containing subpopulations. Original flow cytometry plots are shown for all cell types. Data points are measurements from individual animals; one-way ANOVA with Tukey’s multiple comparison test or respective non-parametric test (**b**–**f**). ^*^P < 0.05 vs. control; ^#^*P* < 0.05 vs. noise

We sought to characterize the seemingly opposing central and peripheral immune responses following noise exposure and DTX treatment. Gating on live, single cells, we were able to discern immune cell types based on their expression of CD45 and CD11b. Namely, microglia are CD45^intermediate^ CD11b^+^, peripheral myeloid populations are CD45^high^ CD11b^+^ and peripheral lymphoid populations are CD45^high^ CD11b^−^ (Fig. 8a). We found significant increases in three markers of microglial activation (CD68, CD86, MHC-II) in Noise, DTX, and DTX + Noise groups, indicating an active central immune response to both noise and DTX treatment (Fig. 8b). Furthermore, we found infiltration of both lymphoid and myeloid peripheral cells in the brains of noise- and especially DTX-treated mice (Fig. 8c, d). Further analysis revealed these myeloid cells to be bone-marrow-derived monocytes and macrophages (Fig. 8e, f), implying either microglial signaling to the peripheral immune system, disruption of the blood–brain barrier, or failure of ablation of the few resident peripheral cells in brain tissue.

## Discussion

To analyze the cardiovascular consequences of aircraft noise with respect to inflammation, oxidative damage, endothelial dysfunction, and hypertension, we used the LysMCre^iDTR^ model to ablate subsets of inflammatory monocytes and macrophages, a system previously used to demonstrate the role of myeloid cells in the development of angiotensin-II-induced hypertension [32, 68]. Our data indicate that myelomonocytic cells are crucially important in the development of noise-induced cardiovascular consequences. Mechanistically, ablation of LysM^+^ cells prevents noise-induced vascular oxidative stress, eNOS uncoupling, endothelial dysfunction, and arterial hypertension. Importantly, comparable effects were not observed in the brain upon LysM^+^ cell ablation. Neither number nor activation of microglia (envisaged by Iba-1 staining and flow cytometry), the major immune cell type in the brain, was decreased following DTX treatment, most likely due to low expression levels of LysM in these cells [52]. Brains of mice who were treated with DTX showed increased markers of inflammation, activation markers of microglia and infiltration of peripheral immune cells as well as higher serum levels of stress hormones (adrenaline, noradrenaline, and corticosterone), indicating that these mice have more pronounced stress responses despite the ablation of LysM^+^ cells in the circulation. The dissociation of the results with persistence of brain inflammation in contrast to anti-inflammatory protection of the vasculature indicates an unaltered cerebral stress response to noise, but also supports the notion that vascular infiltration with inflammatory monocytes and macrophages represents the key event in noise-induced cardiovascular damage.

Much evidence has linked chronic low-grade inflammation to cardiovascular disease, which is now accepted as a risk factor itself [28, 29]. In the CANTOS trial, vascular inflammation was identified as a potential target to treat atherosclerotic disease and treatment of inflammation through monoclonal targeting of IL-1β improved the incidence rate of cardiovascular events dose-dependently without reduction of lipid levels [58]. These results were supported by data the COLCOT study indicating that anti-inflammatory colchicine treatment decreased the risk of ischemic cardiovascular events in patients with a recent myocardial infarction [64]. Genome-Wide Association Studies (GWAS) have also established several risk loci that are linked to cardiovascular inflammation [25, 31], supporting the body of evidence that inflammation is a potent trigger of cardiovascular disease. Mechanistically, increased leukocyte extravasation into the subendothelial space leads to a pro-oxidant and pro-inflammatory milieu. Alterations in the redox balance facilitate the development of endothelial dysfunction and subsequently, dysregulated vascular tone and atherosclerosis as described for cardiovascular risk factors such as smoking or arterial hypertension [35, 68]. Recent studies suggest that noise, which activates similar proinflammatory pathways in the vasculature as "established" cardiovascular disease triggers, should also be considered as a cardiovascular risk factor [35, 39, 43, 68].

NFκB activation and IL-1β induction has been shown to have effects on hypothalamic–pituitary–adrenal (HPA) axis-mediated corticosterone response [27]. From the opposing viewpoint, an immune challenge has also been demonstrated to raise cortisol levels in pigs [66], indicating that stress, inflammation, and cardiovascular disease share overlapping pathomechanisms. In the present work, we show exacerbated NFkB-NLRP3-TXNIP-IL-6 signaling in the brain of DTX-treated mice, which, however, was not translated to the peripheral vasculature as inflammation and impaired vascular function in response to noise was prevented by ablation of LysM^+^ cells.

Experimental and epidemiological studies [39, 59, 61] as well as extensive reviews [2, 3, 8] have connected the noise-induced activation of the HPA axis and sympathetic nervous system along with the release of stress hormones, such as cortisol and catecholamines. The pro-inflammatory effects of mental stress are also well-documented, in which IL-6, IL-1β, and proinflammatory monocytes play a role [38, 72]. In subjects without cardiovascular disease or active cancer, ^18^F-fluorodeoxyglucose positron emission tomography/computed tomography imaging showed that higher noise exposure was associated with higher amygdala activity and vascular inflammation [45, 53]. In the same study, mediation analysis associated major adverse cardiovascular events (MACE) with a serial mechanism involving heightened activity in the amygdala and subsequent arterial inflammation. SAPALDIA, a Swiss cohort study, found independent DNA methylation patterns associated with source-specific exposure to transportation noise and air pollution as well as shared enrichments for pathways related to inflammation, cellular development, and immune responses [13]. Additional insight from targeted proteomic analysis reveals that physiological responses due to exposure to nocturnal train noise included significant changes within redox, pro-thrombotic, and pro-inflammatory pathways [24]. Although direct evidence on immune cell subset changes by noise exposure is not available so far, transcriptome analysis of monocytes from mice and men support a selective up-regulation of a subpopulation of immature proinflammatory monocytes (Ly-6c^(high)^ in mice, CD16^(−)^ in humans) in response to chronic social stress [55], a condition that shares similar stress responses with noise exposure [9].

We would like to add the note of caution that stress hormones are under control of the circadian clock and show substantial in-day variation [71], requiring a strict time protocol for all studies investigating these parameters. Nocturnal noise exposure causes sleep disorders and associated changes in circadian rhythm in humans [12] as well as mice [34]. Accordingly, chronic cortisol increases in the first half of the night, as a read-out for disturbed circadian control of this stress hormone, were described in children with higher nocturnal traffic noise exposure [26].

Translational studies have supported the hypothesis that stress responses resulting from noise exposure result in the induction of inflammatory processes via oxidative stress. Rats exposed to white noise were found to have mesenteric microvascular structural damage resulting in an increased number of leaks. This damage was also seen to be mitigated by treatment with anti-inflammatory and antioxidant co-treatment, supporting an important mechanistic role in the redox and inflammatory processes that result following noise exposure [4]. Evidence from animal studies indicated noise exposure to be associated with altered DNA methylation [20] and telomere length [37], both well-known to be sensitive to inflammation and oxidative stress as well as to predict cardiovascular disease in humans [1, 23]. In previous studies with aircraft-noise exposed mice, we have demonstrated an increase in stress hormones, blood pressure, and oxidative stress in the vasculature and brain. Coupled with findings of increases in leukocyte infiltration in the aortic endothelium, we were able to discern that the oxidative stress originated mostly from phagocytic NOX-2 [34, 39]. In the context of the present study, NOX-2 is constitutively present on LysM^+^ cells, indicating that these cells are an important factor in the generation of oxidative stress in response to noise-derived stress. In the brain, we previously found a neuroinflammatory phenotype characterized by astrocyte activation and inflammatory markers alongside increases in oxidative stress, and was worsened by the presence of pre-existing hypertension [34, 63]. These deleterious effects were almost completely prevented in *Nox2* knockout mice, confirming a crucial role for these cells in the detrimental phenotype resulting from acute noise exposure [34].

Here, we expand our understanding of the neuroinflammatory phenotype induced by noise by demonstrating a clear involvement of microglia, the major immune cell of the brain. As tissue resident macrophages, microglia also express NOX-2 and produce ROS in a wide variety of pathologies in both retinal and cerebral tissues [14, 56, 76]. Interestingly, the retina contains both LysM^+^ peripheral macrophages and central microglia [36], offering a possible explanation for the partial normalization of endothelial function and ROS generation we recorded in the retinal vessels of this study. Though we were unable to measure endothelial function of cerebral vessels, this partial normalization yields some insight to the effects of microglial activation on vessel function. Furthermore, we delineate that LysM^+^ cells in the peripheral vessels (aorta and mesentery) are largely responsible for the generation of damaging oxidative stress following noise exposure and its subsequent pathomechanisms.

Our present findings may also be applicable to a broader range of mental stress conditions. Similar to many other psychological stressors such as the chronic experience of negative emotions (e.g., grief, fear/panic, anger, anxiety, or embarrassment) in connection with stressful (life) events (e.g., death of a relative/friend, divorce, family conflict, or job loss), chronic noise stress can result in cumulative adverse health consequences and in particular in cardiovascular diseases by triggering several disease-promoting physiological changes, including hypothalamic–pituitary–adrenal axis activation, behavioral and cardiometabolic changes, increased sympathetic nervous system and decreased parasympathetic nervous system activity, heightened leukopoiesis, and immune dysregulation [54]. However, it is important to acknowledge that unlike many other psychological stressors, noise is ubiquitous globally and can hardly be avoided or improved upon by lifestyle choices and thus cannot be controlled by oneself and others.

As a limitation of the study, we note that assessment of vascular function of cerebral microvessels was not included, which could have clarified the impact of the activated microglia and infiltrating peripheral immune cells on cerebral microvascular function. It should be noted that retinal vessels are part of the central nervous system, sharing similarities with cerebral vessels such as an endothelial barrier (blood–brain and blood–retinal barrier), organization in a neurovascular unit, the presence of pericytes, autoregulation, and the correlation between pathomechanisms of cerebral small vessel disease and retinal vascular abnormalities [6, 7, 46]. However, there are also significant differences between retinal and cerebral vessels regarding anatomy and physiology such as vascular acetylcholine responses in mice mediated by M_5_ receptors in cerebral vessels [73] but by M_3_ receptors in retinal vessels [18]. Moreover, retinal and cerebral vessels differ with regard to their pericyte-to-endothelial cell ratio [62]. Cochlear vascular function was also not assessed, which may represent another limitation of our study, as cochlear inflammation and oxidative stress by noise exposure (even when below the threshold of hearing loss) may propagate to the general circulation and cause peripheral vascular inflammation, oxidative stress and dysfunction.

## Conclusions

**Fig. 9:**
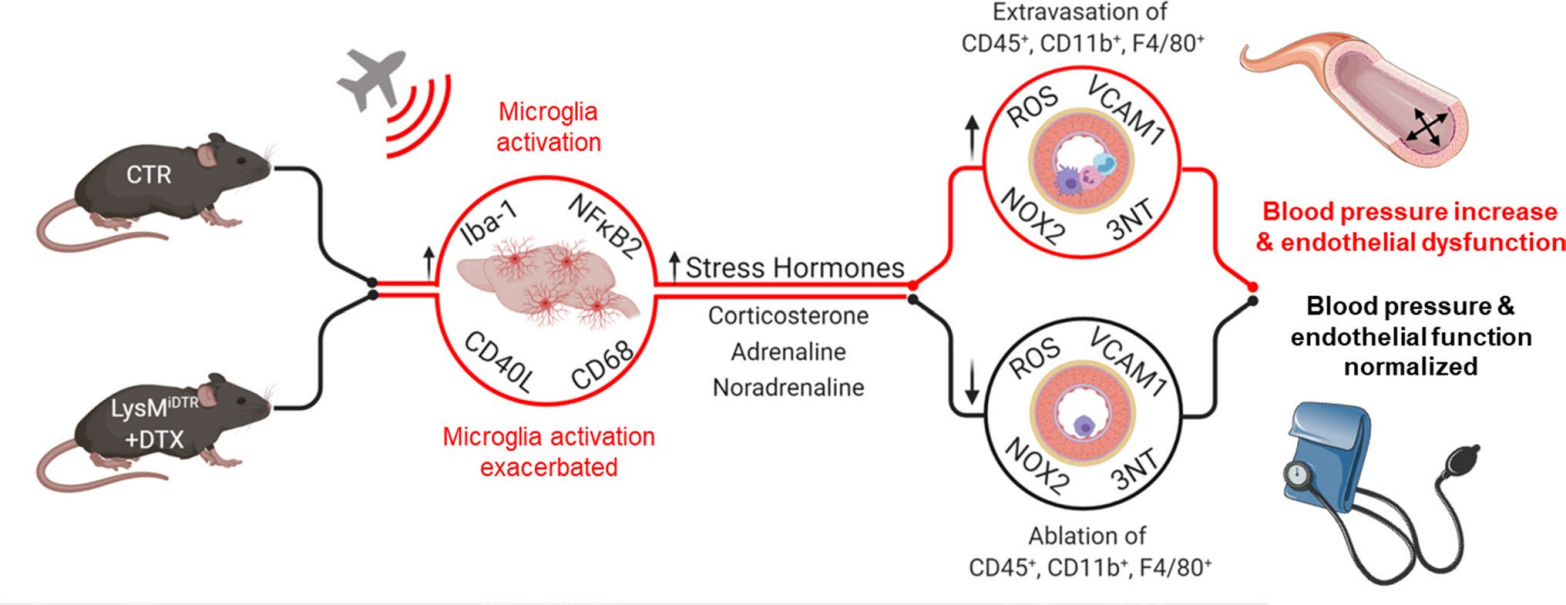
Summary scheme. Noise-induced stress responses were not prevented by DTX treatment as the central immune cells, microglia, were unablated and activated under these conditions. Stress hormone release was also exacerbated in LysMCre^iDTR^ mice with DTX treatment. Nevertheless, LysM^+^ cell ablation prevented aircraft noise-induced endothelial dysfunction, oxidative stress, inflammation and blood pressure elevation. These data demonstrate that infiltration of the vasculature by LysM^+^ cells is crucial for noise-induced cardiovascular damage and that central immune responses via microglia are important mediators in these actions. Created with BioRender.com

The present study used the LysMCre^iDTR^ / DTX model for specific ablation of LysM^+^ cells to demonstrate that infiltration of the vasculature by LysM^+^ cells is crucial for aircraft noise-induced endothelial dysfunction, oxidative stress, inflammation and blood pressure elevation (Fig. 9) and also that these cells may be centrally recruited and exacerbate neuroinflammation. Noise-induced stress responses and central immune responses were unchanged or heightened by DTX treatment, as microglia were highly activated and considerable peripheral immune infiltration was found in the brains of exposed mice, but were not translated into peripheral cardiovascular consequences. Our data indicate that the suppression of vascular infiltration of LysM^+^ cells is sufficient to prevent noise-induced stress hormone-dependent macro- and microvascular dysfunction peripherally, but also that these effects may arise from central oxidative stress, inflammation, or stress responses. These results may imply that treatment of cardiovascular symptoms requires central and peripheral medication to mitigate the true extent of noise-induced pathology. Thus, besides established cardiovascular risk factors, it becomes more and more evident that also new environmental factors such as noise contribute significantly to the burden of cardiovascular disease [10, 44]. Accordingly, the environmental stressor noise should be mentioned in the guidelines for the prevention of cardiovascular disease next to diabetes, smoking, arterial hypertension, and hyperlipidemia.

## Abbreviations

ACh: Acetylcholine
dB(A): Decibel
DHE: Dihydroethidium
DTX: Diphtheria toxin
GFAP: Glial fibrillary acidic protein
KH: Krebs–Henseleit
L-NAME: N^G^-nitro-l-arginine methyl ester
LysM: Lysozyme M
NFκB: Nuclear factor-κB
NGS: Normal goat serum
NHS: Normal horse serum
NIBP: Non-invasive blood pressure
NLRP3: NLR family pyrin domain containing 3
eNOS: Endothelial nitric oxide synthase
NOX: NADPH oxidase
NOX-2: Phagocytic NADPH Oxidase
NTG: Nitroglycerin
ROS: Reactive oxygen species
rtPCR: Real-time PCR
TXNIP: Thioredoxin interacting protein
VCAM-1: Vascular cell adhesion molecule-1
